# Increased Osteoblast Gα_S_ Promotes Ossification by Suppressing Cartilage and Enhancing Callus Mineralization During Fracture Repair in Mice

**DOI:** 10.1002/jbm4.10841

**Published:** 2023-11-15

**Authors:** Kathy K Lee, Adele Changoor, Marc D Grynpas, Jane Mitchell

**Affiliations:** ^1^ Department of Pharmacology and Toxicology University of Toronto Toronto Canada; ^2^ Lunenfeld‐Tanenbaum Research Institute Mount Sinai Hospital Toronto Canada; ^3^ Department of Surgery University of Toronto Toronto Canada; ^4^ Department of Laboratory Medicine and Pathobiology University of Toronto Toronto Canada

**Keywords:** BONE REPAIR, CANONICAL WNT SIGNALING, ENDOCHONDRAL BONE FORMATION, Gα_S_ SIGNALING, OSTEOBLAST DIFFERENTIATION

## Abstract

Gα_S_, the stimulatory G protein α‐subunit that raises intracellular cAMP levels by activating adenylyl cyclase, plays a vital role in bone development, maintenance, and remodeling. Previously, using transgenic mice overexpressing Gα_S_ in osteoblasts (G_S_‐Tg), we demonstrated the influence of osteoblast Gα_S_ level on osteogenesis, bone turnover, and skeletal responses to hyperparathyroidism. To further investigate whether alterations in Gα_S_ levels affect endochondral bone repair, a postnatal bone regenerative process that recapitulates embryonic bone development, we performed stabilized tibial osteotomy in male G_S_‐Tg mice at 8 weeks of age and examined the progression of fracture healing by micro‐CT, histomorphometry, and gene expression analysis over a 4‐week period. Bone fractures from G_S_‐Tg mice exhibited diminished cartilage formation at the time of peak soft callus formation at 1 week post‐fracture followed by significantly enhanced callus mineralization and new bone formation at 2 weeks post‐fracture. The opposing effects on chondrogenesis and osteogenesis were validated by downregulation of chondrogenic markers and upregulation of osteogenic markers. Histomorphometric analysis at times of increased bone formation (2 and 3 weeks post‐fracture) revealed excess fibroblast‐like cells on newly formed woven bone surfaces and elevated osteocyte density in G_S_‐Tg fractures. Coincident with enhanced callus mineralization and bone formation, G_S_‐Tg mice showed elevated active β‐catenin and Wntless proteins in osteoblasts at 2 weeks post‐fracture, further substantiated by increased mRNA encoding various canonical Wnts and Wnt target genes, suggesting elevated osteoblastic Wnt secretion and Wnt/β‐catenin signaling. The G_S_‐Tg bony callus at 4 weeks post‐fracture exhibited greater mineral density and decreased polar moment of inertia, resulting in improved material stiffness. These findings highlight that elevated Gα_S_ levels increase Wnt signaling, conferring an increased osteogenic differentiation potential at the expense of chondrogenic differentiation, resulting in improved mechanical integrity. © 2023 The Authors. *JBMR Plus* published by Wiley Periodicals LLC. on behalf of American Society for Bone and Mineral Research.

## Graphical abstract

In a mouse model of bone fracture repair, increased Gs expression in osteoblasts suppresses chondrocyte differentiation and favors osteoblastic bone formation. Increased Gs stimulated canonical Wnt expression and signaling. Gs‐stimulated bone formation results in greater bone mineral density and improved material stiffness.

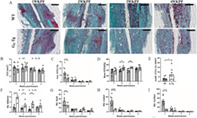

## Introduction

Fractures are the most common type of traumatic injuries requiring hospitalization.^(^ ^1^ ^)^ A non‐healing fracture is typically treated with surgery to restore anatomical configuration and maintain alignment with rigid internal fixation.^(^ ^2^ ^)^ Use of rigid compression plates to tightly maintain alignment promotes intramembranous healing, in which the reconstitution of injured bone is achieved without callus formation through direct differentiation of mesenchymal progenitors into osteoblasts.^(^ ^2^ ^)^ Most fractures, however, repair via endochondral bone healing, which requires initial bridging of the fracture gap by cartilage matrix produced by chondrocytes before bony union by osteoblast.^(^ ^3^ ^)^ Bone repair is complete once disorganized bone matrix is remodeled into structurally and functionally mature lamellar bone.^(^ ^4^ ^)^ The dynamic nature of endochondral bone repair necessitates a well‐orchestrated activation and integration of multiple regulatory pathways that direct differentiation of multipotent mesenchymal progenitors into chondrocytes and into osteoblasts during callus formation. Any failure in these processes leads to malunion of the fracture. Hence, improved understanding of the molecular mechanisms underlying the reparative phases of the fracture healing cascade will be of paramount importance for the development of effective pharmacological treatment that can enhance and accelerate endochondral bone repair.

One such regulatory pathway is G protein α‐subunit (Gα_S_) signaling.^(^ ^5^ ^)^ Loss‐ and gain‐of‐function studies have demonstrated that aberrant Gα_S_ activity in osteoblast lineage cells has profound effects on osteoblast differentiation and activity and thus, bone microarchitecture, density, and mass accrual. Constitutive activation of Gα_S_ in mice recapitulates skeletal manifestations of fibrous dysplasia, including expansion of trabeculae in woven bone, marrow fibrosis, and impaired bone mineralization, owing to maturational arrest of osteoblasts.^(^ ^6^, ^7^ ^)^ Similarly, cortical bone loss and increased formation of disorganized trabecular bone admixed with fibrotic cells indicative of aberrant osteoblast differentiation is observed in mice expressing a constitutively active mutant PTH1R.^(^ ^8^ ^)^ Inactivating mutations of Gα_S_, in contrast, are associated with pseudohypoparathyroidisim type 1a, and its deficiency in murine osteoblastic cells compromises osteoblast differentiation and trabecular bone formation.^(^ ^9^ ^)^

Animal studies demonstrating the osteoanabolic actions of parathyroid hormone (PTH) and prostaglandin E_2_ (PGE_2_) that bind to G protein–coupled receptors (GPCRs) also implicate the involvement of Gα_S_ signaling in regulating endochondral bone repair. Intermittent PTH treatment in mice delays full replacement of cartilage anlagen by endochondral bone during skeletal repair due to the inhibitory effect of PTH on chondrocyte maturation, yet it improves biomechanical properties by enhancing osteoblastogenesis and new bone formation.^(^ ^10^ ^)^ Similar alterations, but with a delay in the onset of peak bony callus, are observed with continuous infusion of PTH.^(^ ^11^, ^12^ ^)^ The osteostimulatory effect of PTH on bone fractures in humans is also evidenced by decreased time to bony union in several clinical studies.^(^ ^13^, ^14^ ^)^ Additionally, stimulation of cAMP production via Gα_S_ by a selective EP4 receptor agonist enhances osteogenic response, leading to increased bone formation during endochondral repair.^(^ ^15^, ^16^ ^)^ Together, these findings illuminate the importance of the right level of Gα_S_ activity in promoting endochondral ossification during bone repair. There is accumulating evidence that bone cells are also sensitive to variation in normal Gα_S_ levels. In vitro studies with osteoblastic cells have shown that drugs with known influence on bone, such as retinoic acid and glucocorticoids, modulate the abundance of Gα_S_ within cells and, consequently, the activity of its major downstream effector adenylyl cyclase.^(^ ^17^, ^18^ ^)^ Furthermore, Yang and colleagues provided evidence for natural differences in Gα_S_ levels in various human cells in a study that reported a 3‐ to 4‐fold range in Gα_S_ expression among healthy individuals with corresponding changes in intracellular cAMP levels.^(^ ^19^ ^)^ Previous work in our lab provided further insight into the in vivo effects of variation in Gα_S_ levels within the bone milieu using transgenic mice with osteoblast‐specific overexpression of Gα_S_ (Gs‐Tg). Skeletal overexpression of the Gα_S_ in the transgenic mice was previously assessed both at the mRNA level by qPCR using primers that recognize total Gα_S_ (Gα_S_ short and long isoforms) and protein level by Western blotting. In femoral trabecular bone of G_S_‐Tg mice, an average of 5‐fold and 4.6‐fold increase in mRNA and protein expression were detected, respectively.^(^ ^20^, ^21^ ^)^ G_S_‐Tg mice presented increased trabecular bone mass due to a preferential increase in osteoblast activity but reduced bone quality owing to increased cortical porosity and formation of disorganized woven bone.^(^ ^20^ ^)^ High levels of Gα_S_ also altered osteoblastic response to hyperparathyroidism (HPT). Continuous exposure to PTH resulted in greater cAMP production in osteoblasts in vitro and increased trabecular bone volume with peritrabecular fibrosis in vivo in contrast to trabecular bone loss in wild‐type (WT) mice, as observed in patients with HPT.^(^ ^21^ ^)^ These studies point to the importance of precise regulation of Gα_S_ levels in osteoblasts, adding additional layer of complexity to G protein–mediated regulation of skeletal development and homeostasis.

Given that the regenerative processes of fracture healing recapitulate embryonic endochondral ossification, we hypothesize that Gα_S_ levels will influence bone repair. However, increased *GNAS* and cAMP signaling components were only one of several pathways associated with mouse strain–specific differences in endochondral bone repair.^(^ ^22^ ^)^ It is not known if increased osteoblast Gα_S_ alone is sufficient to alter the fracture repair process. The aim of this study is therefore to investigate the effects of osteoblast Gα_S_ overexpression on bone regeneration and remodeling during fracture healing and to explore the underlying molecular mechanisms using a tibial osteotomy model for endochondral bone healing.

## Materials and Methods

### Animals

Gα_S_ transgenic mice (G_S_‐Tg) expressing *GNAS* encoding human Gα_S_‐long subunit under the control of the 3.6‐kb rat Col1a1 promoter were generated on the FVB background strain as previously described.^(^ ^20^ ^)^ All mice were bred and maintained under standard housing conditions with free access to rodent chow and tap water. All animal procedures were reviewed and approved by the animal care committee of the University of Toronto.

Only male mice were used because of practical difficulties in inserting the intramedullary pin in the narrow medullary canal of the tibia in female mice. No apparent sexual dimorphism in basal trabecular and cortical bone phenotypes is observed in G_S_‐Tg mice.^(^ ^20^ ^)^ Sixty‐three mice were used for micro‐CT evaluation followed by histology at 1, 2, 3, and 4 weeks post‐fracture (*n* = 6–10 mice/group) and immunohistochemistry at 2 weeks post‐fracture (*n* = 3 mice/group). A separate group of 53 mice were euthanized at 1, 2, 3, and 4 weeks post‐fracture for RT‐qPCR (*n* = 5–8 mice/group). For biomechanical testing at 4 weeks post‐fracture, an additional 17 mice were euthanized (*n* = 8–9 mice/group).

### Osteotomy model

Unilateral open diaphyseal fractures were produced in the left tibias of 8‐week‐old male wild‐type and G_S_‐Tg mice as previously described.^(^ ^23^, ^24^ ^)^ After anesthesia with isoflurane (2–3% inhalation), the left hind leg was shaved and disinfected with iodine and 75% EtOH. A small skin incision was made at the knee joint, and intramedullary fixation was carried out by inserting a sterile insect pin down the medullary canal through an entry hole in the tibia plateau created with a 24G syringe needle. Subsequently, a transverse osteotomy was performed slightly above the tibial midshaft with surgical scissors and the incision was closed with sutures and metallic wound clips to prevent the mouse from removing stitches and displacing the pin. Mice received meloxicam (2 mg/kg s.c.) and extended‐release buprenorphine (1 mg/kg s.c.) preoperatively and meloxicam only for an additional 2 days after the operation for pain relief. There is conflicting data on the negative effects of NSAIDs, such as meloxicam, on fracture healing.^(^ ^25^, ^26^, ^27^, ^28^ ^)^ Nonetheless, given the relative short half‐life of meloxicam in mice (4–6 hours) and the long interval between doses (once daily), there should be daily periods during which COX‐2‐dependent synthesis of prostaglandin can still occur.

Upon recovery from anesthesia, mice were housed individually with free access to food and water and permitted to ambulate freely and bear weight as tolerated. Bone fractures under these conditions repair through endochondral ossification in the center of the fracture site and intramembranous ossification in the distal edges of the callus.^(^ ^29^ ^)^ To ensure consistency in fracture quality, X‐rays of the fractured tibias were taken before euthanasia to exclude any animals with displaced pins and misaligned fractures from subsequent analyses. Example X‐ray images at 1 week post‐fracture are shown in Supplemental Fig. S1. Mice were weighed and euthanized by CO_2_ inhalation followed by cervical dislocation.

### Micro‐CT examination

Both fractured and intact contralateral tibias were harvested at 1, 2, 3, and 4 weeks post‐fracture by carefully trimming off surrounding muscle and soft tissue and fixed in 10% neutral‐buffered formalin overnight before removal of the intramedullary pin without disturbing the callus. Bones were scanned using micro‐CT (Skyscan 1174, Bruker, Kontich, Belgium) with an isotropic voxel size of 11.6 μm^3^, an integration time of 3800 ms, an X‐ray tube voltage of 50 kV, a current of 800 mA, and a 0.25 mm aluminum filter used for image acquisition. A set of manufacturer‐provided hydroxyapatite (HA) phantoms (250 mg and 750 mg HA/cm^3^) were scanned daily to calibrate vBMDs.

Image reconstruction and morphometric analyses were conducted using NRecon and CTAn (versions 1.7.4.6 and 1.18.8.0, Skyscan, Bruker), respectively. For each fractured tibia, the fracture line was identified in the sagittal plane (Dataviewer, Skyscan) and 250 axial slices centered on the fracture line was defined as the volume of interest (VOI). The outer boundary of the callus encompassing all tissues (bone, cartilage, and void) was manually delineated in the 2D tomograms between the proximal and distal boundaries of the callus to define the total callus volume (TCV). Callus was segmented into either newly mineralized tissue or bone based on the density of the intact cortical bone. The maximum density of intact cortical bone was determined by analyzing 250 slices of the contralateral tibia centered in the mid‐diaphyseal region that is spatially coincident with the fracture site. A threshold range corresponding to 35% to 57% of the contralateral maximum cortical density was applied to segment mineralized callus volume (MCV). Tissues with densities below 35% were considered non‐mineralized callus. Total mineralized tissue volume, which includes newly mineralized tissue and bone, was normalized to callous volume (TMV/TCV, %) using a threshold of 35% to 100% of maximum cortical density. Bone volume normalized to callus volume (BV/TCV, %) was measured using a threshold of 57% to 100% of the maximum cortical density. These threshold ranges were selected with reference to a previous publication^(^ ^30^ ^)^ and through visual comparisons.

### Histology and histomorphometric analyses

After micro‐CT scanning, the specimens were fixed in 10% formalin for an additional 2 days, decalcified in 0.5 M EDTA solution, pH 7.4, at room temperature (RT) for 5 days, and embedded in paraffin. Serial sagittal sections (5 μm thick) were stained with Safranin‐O/Fast Green to identify cartilage and bone and tartrate‐resistant acid phosphatase (TRAP) (manufacturer's protocol, 387A‐1KT, Sigma, St. Louis, MO, USA) to identify osteoclasts. Histomorphometric analyses were performed using BioQuant software (version 21.5.6, BIOQUANT Image Analysis Corporation, Nashville, TN, USA). The region of interest (ROI) was determined by manually tracing around the region of callus extending 1 mm proximally and distally from the center of the osteotomy gap defined by the maximum callus width. The relative proportions of cartilage and bone were determined by quantifying the areas of Safranin‐O‐positive proteoglycan (red) and Fast Green‐positive collagen (blue), respectively, via a combination of thresholding and manual editing of the selected region and expressing as the percentage of the total callus area.

Histological analysis of fibroblasts, osteoblasts, and osteocytes was based on their characteristic morphology and location within the callus. Fibrosis volume per tissue volume (FV/TV, %) was defined as the volume of extracellular matrix–containing cells with spindle‐shape appearance located adjacent to the bone perimeter as a percent of tissue volume. For quantification of osteoblasts, osteoblast surface was reported as the percentage of bone surface covered by plump cuboidal cells (Ob.S/BS, %). Osteocytes, normalized to either tissue volume (N.Ot/TV, 1/mm^2^) or bone volume (N.Ot/BV, 1/mm^2^), were identified as cells embedded in lacunae in the bone matrix. The formation and activity of osteoclasts were determined by quantifying the number and surface of TRAP‐positive multinucleated (≥3 nuclei) cells found adjacent to bone surfaces.

For quantification of callus tissue composition (cartilage, bone, undifferentiated tissue) and osteoclast number and surface, whole callus was analyzed in each mouse. For histomorphometric measurements of osteoblasts, fibrosis, and osteocytes, three fields of view (FOV) within each callus section from *n* = 5 animals per genotype per time point were used. The mean of 3 FOVs represented one sample and was used in statistical analysis.

### RNA extraction and real‐time PCR

For RNA analyses, fractured and unfractured contralateral tibias were dissected free of soft tissue at 1, 2, 3, and 4 weeks post‐fracture. After removing the intramedullary pin, a 5‐ to 6‐mm region encompassing the entire fracture callus was cut and snap‐frozen in liquid nitrogen. Frozen samples were pulverized with a prechilled mortar and pestle and further homogenized in 1 mL Trizol (Life Technologies, Burlington, Canada). Total RNA was purified from homogenized tissue via phase separation according to the manufacturer's protocol. Each RNA sample was prepared from tibias pooled from two mice to ensure sufficient RNA yield. One microgram of RNA was treated with DNase I (Life Technologies) and reverse transcribed into cDNA using M‐MLV reverse transcriptase (Life Technologies). All qPCR reactions were performed in triplicate using PowerUp SYBR master mix (Life Technologies) and β2‐macroglobulin as the endogenous control in a QuantStudio 3 real‐time PCR system (Applied Biosystems–Thermo Fisher, Mississauga, ON, Canada). Relative expression levels of each gene were calculated with respect to 1WKPF wild‐type intact contralateral bone. Primers were designed using Primer‐BLAST (National Center for Biotechnology Information [NCBI], Bethesda, MD, USA) and are listed in Supplemental Table S1.

### Immunofluorescence staining

Two‐week‐old fracture calluses were harvested, formalin fixed for 24 hours at RT, and decalcified in 0.5 M EDTA (pH 7.4). Decalcified specimens were processed, embedded in paraffin, and sectioned sagitally at a thickness of 5 μm. Tissue sections were baked for 15 minutes at 60°C to soften the wax, deparaffinized in xylene, and rehydrated in a series of graded alcohols. Heat‐induced antigen retrieval was performed in 10 mM sodium citrate buffer (pH 6.0) at 95°C for 15 minutes. Subsequently, the sections were blocked with 3% BSA in PBS containing 0.3% Triton X‐100 (Sigma‐Aldrich, Oakville, Canada) in a humidified chamber for 1 hour at RT and incubated with primary antibody overnight at 4°C. Rabbit anti‐non‐phospho‐β‐catenin monoclonal antibody (1:200, #8814, Cell Signaling Technologies, Whitby, Canada) and anti‐Wntless/GPR177 polyclonal antibody (1:200, #17950‐1‐AP, Thermo Fisher Scientific) were used as primary antibodies and were diluted in PBS containing 1% BSA and 0.1% Triton X‐100. For negative control sections, the primary antibodies were replaced with an isotype‐matched antibody (#NI01, Sigma‐Aldrich). Then, after four 5‐minute PBS washes, slides were incubated with Alexa Fluor 647‐conjugated secondary antibody (1:250 for β‐catenin and 1:500 for Wntless; Thermo Fisher Scientific) for 1 hour at RT and rinsed again in PBS. The sections were incubated with TrueVIEW Autofluorescence Quenching Reagent to reduce autofluorescence and mounted with VECTASHIELD Vibrance Mounting Medium with DAPI (Vector Laboratories, Burlingame, CA, USA). Fluorescence images were acquired with a spinning disc confocal microscope (Quorum Technologies, Puslinch, Canada).

### Torsion testing

Before biomechanical testing, the specimens were imaged using micro‐CT to obtain callus bone morphometric parameters: total mineral density (TMD), total mineral content (TMC), total mineralized tissue volume (TMV), cortical thickness, cross‐sectional bone area, maximum and minimum radii, and polar moment of inertia. The torsional biomechanical properties of the healing tibias at 4 weeks post‐fracture were evaluated using a Mach‐1 Mechanical Tester (Biomomentum Inc., Laval, Canada) (*n* = 8–9 per group). Each fracture sample was thawed at room temperature for 1 hour and any residual soft tissue was removed. A 4‐mm gauge length was marked on each specimen by first identifying the original osteotomy site in the longitudinal micro‐CT views through visual inspection and measuring 2 mm on either side of the fracture site. The proximal and distal ends were potted in disposable fixtures filled with polymethylmethacrylate (PMMA). The proximal end of the specimen was centrally positioned within the fixture, but to ensure the gauge length was vertically aligned between the mounts and accommodate the natural curvature of the tibia, the distal end was necessarily off‐centered in its mount. Immediately before testing, samples were immersed for a minimum of 20 minutes in phosphate buffered saline. Each specimen was preconditioned with 10 nondestructive cycles of ±5^o^ at 0.1 Hz and then subjected to torque to failure at a rate of 1^o^/s. Whole‐bone mechanical properties, including yield torque and twist to failure, were determined from the torque (T) and angular displacement (^o^) curves. Torsional stiffness was defined as the slope of the linear portion of the curve and energy‐to‐failure as the area beneath the curve up until the yield torque. Tissue mechanical properties, such as shear stress, shear strain, shear modulus, and toughness, were calculated by normalizing to the polar moment of inertia, gauge length, and volume of bone tested.

### Statistical analysis

Data are presented as the means ± standard error (SEM), unless otherwise stated, with the sample numbers indicated in the figure legends. To compare differences among the genotypes across time points in micro‐CT, histology, and gene expression analyses, two‐way ANOVA followed by Sidak's multiple comparisons test was performed using GraphPad Prism 8 (GraphPad, La Jolla, CA, USA). For statistical evaluation of differences between WT and Gs‐Tg samples in undifferentiated tissue volume fraction, osteoclast parameters, osteoclast marker expression, and mechanical testing data, unpaired *t* tests were carried out at each time point. For qualitative analysis of immunostaining data, two FOVs per animal and three animals from each genotype were used, and the representative results were presented. Differences were considered significant at *p* < 0.05.

## Results

### Osteoblastic Gs overexpression enhances callus mineralization and bone formation but reduces cartilage formation

**Fig. 1:**
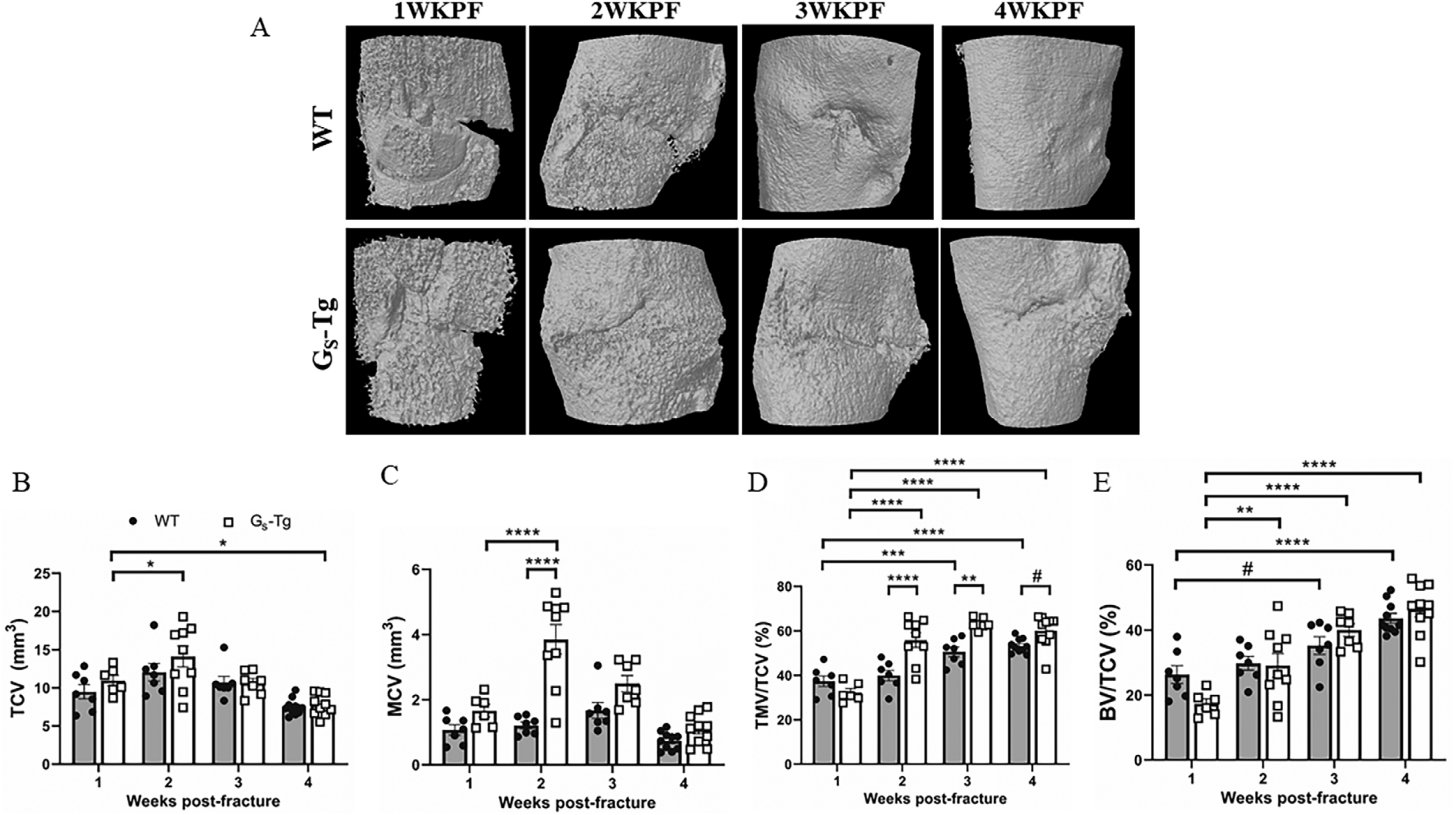
High levels of Gα_S_ increase callus mineralization. (*A*) Representative longitudinal micro‐CT images of fractured tibias of wild‐type (WT) and G_S_‐Tg mice. (*B–E*) Quantification of callus volume and mineralization at 1 (WT, *n* = 7; G_S_‐Tg, *n* = 6), 2 (WT, *n* = 7; G_S_‐Tg, *n* = 9), 3 (WT, *n* = 7; G_S_‐Tg, *n* = 7), 4 (WT, *n* = 10; G_S_‐Tg, *n* = 10) weeks post‐fracture. (*B*) TCV = total callus volume; (*C*) MCV = mineralized callus volume; (*D*) TMV/TCV = total mineralized callus volume fraction; (*E*) BV/TCV = bone volume fraction. Values represent mean ± SEM. Two‐way ANOVA with Sidak's post hoc test was used for statistical comparisons. *p < 0.05, ***p < 0.001, ****p < 0.0001, #*p* ≤ 0.1 compared with WT mice.

Osteotomies were produced using fine surgical scissors generating gaps of approximately 0.5 mm and were not significantly different between WT and G_S_‐Tg mice when measured by X‐ray at 1 week post‐fracture (WT 0.49 ± 0.09; G_S_‐Tg 0.48 ± 0.1 mm). To determine if the metal pins inserted into the tibial medullary cavities would offer the same level of stability to fractured bones of both genotypes, the volume of the medullary cavity was measured using micro‐CT in the contralateral intact tibias of WT and G_S_‐Tg mice in the region corresponding to fracture callus in the fractured tibia at 1WKPF and found no significant difference between groups (Supplemental Figs. S1 and S2). Fracture calluses were examined weekly over a 4‐week period by micro‐CT to determine the effects of increased levels of Gα_S_ on the longitudinal changes in callus formation and mineralization (Fig. 1*A–E*). No overt differences in total callus volume between genotypes were observed (Fig. 1*B*). Total mineralized tissue volume normalized to callus volume (Fig. 1*D*) and total normalized bone volume (Fig. 1*E*) increased in WT mice at 2 weeks and was significantly increased at 3 and 4 weeks post‐fracture. However, the rate and amount of mineralized callus tissue formed (Fig. 1*C*) and the total mineralized tissue volume fraction (Fig. 1*D*) were substantially enhanced in G_S_‐Tg mice beginning at 2 weeks post‐fracture. Peak mineralized callus volume occurred at 2 weeks in G_S_‐Tg, by which time ossified tissue predominated the callus in G_S_‐Tg fractures (Fig. 1*C*). When normalized to total callus volume, G_S_‐Tg mice consistently showed a significantly greater percentage of total mineralized callus tissue from 2 to 4 weeks post‐fracture compared with WT (Fig. 1*D*, *p* < 0.05).

**Fig. 2:**
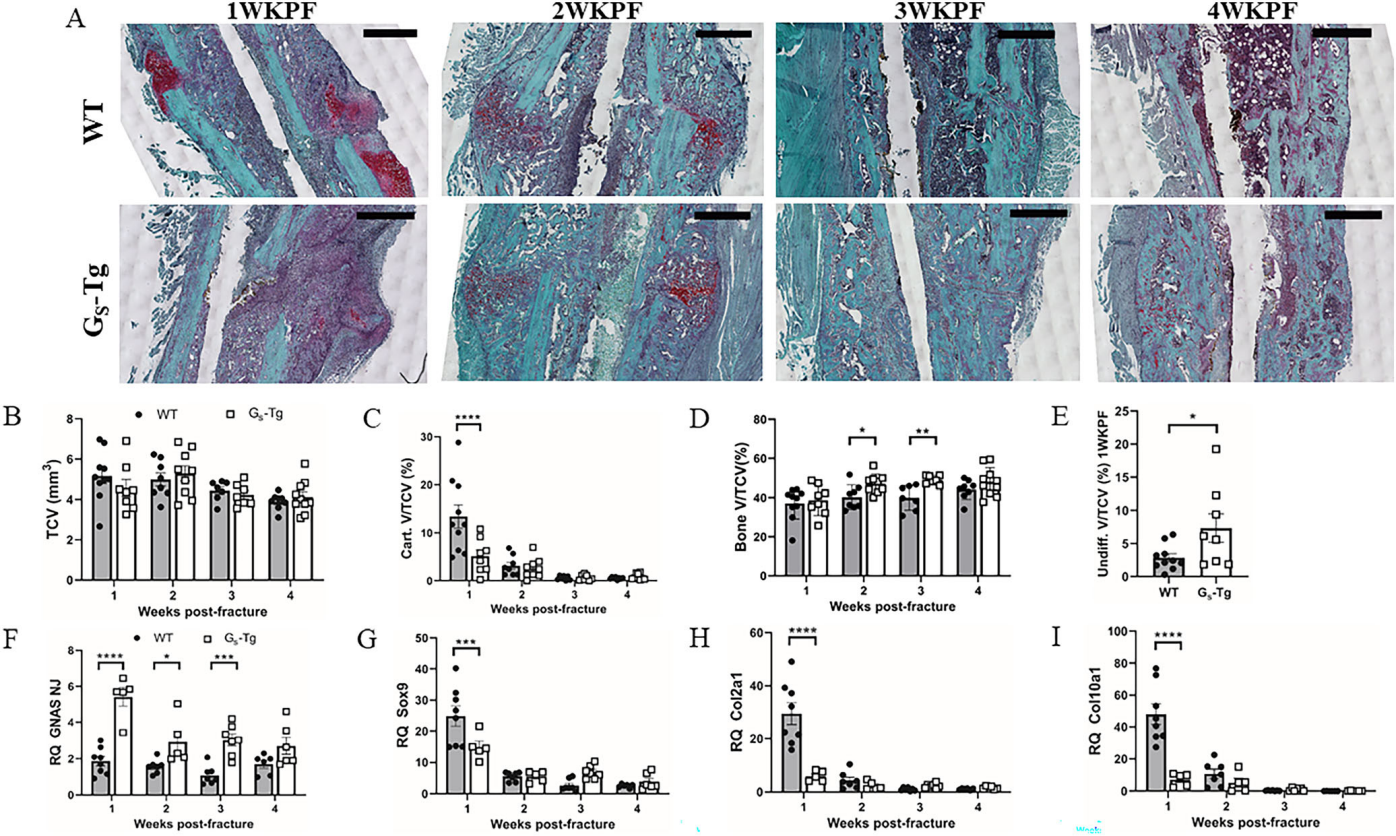
High levels of Gα_S_ enhance bone formation but suppress cartilage formation. (*A*) Representative histological images of Safranin‐O/Fast Green–stained sections of fracture calluses from wild‐type (WT) and G_S_‐Tg mice. Scale bar = 1000 μm. (*B–D*) Histomorphometric quantification of (*B*) total callus volume (TCV); (*C*) cartilage volume fraction (Cart. V/TCV); (*D*) bone volume fraction (Bone V/TCV) at 1 (WT, *n* = 10; G_S_‐Tg, *n* = 8), 2 (WT, *n* = 8; G_S_‐Tg, *n* = 9), 3 (WT, *n* = 7; G_S_‐Tg, *n* = 7), and 4 (WT, *n* = 8; G_S_‐Tg, *n* = 10) weeks post‐fracture and (*E*) undifferentiated volume fraction (Undiff. V/TCV) at 1 week post‐fracture. (*F–I*) RT‐qPCR analysis of mRNA expression of Gα_S_ and chondrocyte‐specific genes: SRY‐box transcription factor 9 (*Sox 9*), type II collagen alpha 1 chain (*Col2a1*), type X collagen alpha 1 chain (*Col10a1*). RT‐qPCR data were normalized to beta 2 microglobulin (β2M) expression and fold changes were expressed relative to the WT contralateral intact tibia at 1 week post‐fracture. Values represent mean ± SEM. Two‐way ANOVA with Sidak's post hoc test was used to detect genotype‐specific significant differences in *B–D* and *F–I*. Student's *t* test was used to compare between groups in *E*. *p < 0.05, **p < 0.01, ***p < 0.001, ****p < 0.0001 compared with WT mice.

To evaluate the proportions of cartilage and bone within the fracture callus, micro‐CT analysis was complemented by histological assessment of Safranin‐O/Fast Green–stained callus tissue sections (Fig. 2*A–E*). Quantitative histomorphometry revealed a 62% reduction in cartilage volume at 1 week in G_S_‐Tg mice compared with wild type (Fig. 2*C*). The reduced histological appearance of cartilage tissue was validated by uniform downregulation of chondrogenic differentiation markers *Sox9*, *Col2a1*, and *Col10a1* at 1 week coincident with peak Gα_S_ transgene expression (Fig. 2*G–I*). Though there was no concurrent change in the relative bone volume at 1 week in G_S_‐Tg (Fig. 2*D*), a significant increase was found in the percentage volume of undifferentiated tissue at 1 week (Fig. 2*E*), followed by 17% and 23% increase in percent bone volume in G_S_‐Tg compared with wild‐type mice at 2 and 3 weeks, respectively (Fig. 2*D*). Taken together, micro‐CT and histological findings indicate that high levels of Gα_S_ promote ossification while suppressing cartilage formation and subsequently enhancing callus mineralization and bony union during the osseous phases of bone repair.

### High levels of G_S_ in osteoblast precursors promotes osteogenesis and fibrosis during bone repair

**Fig. 3:**
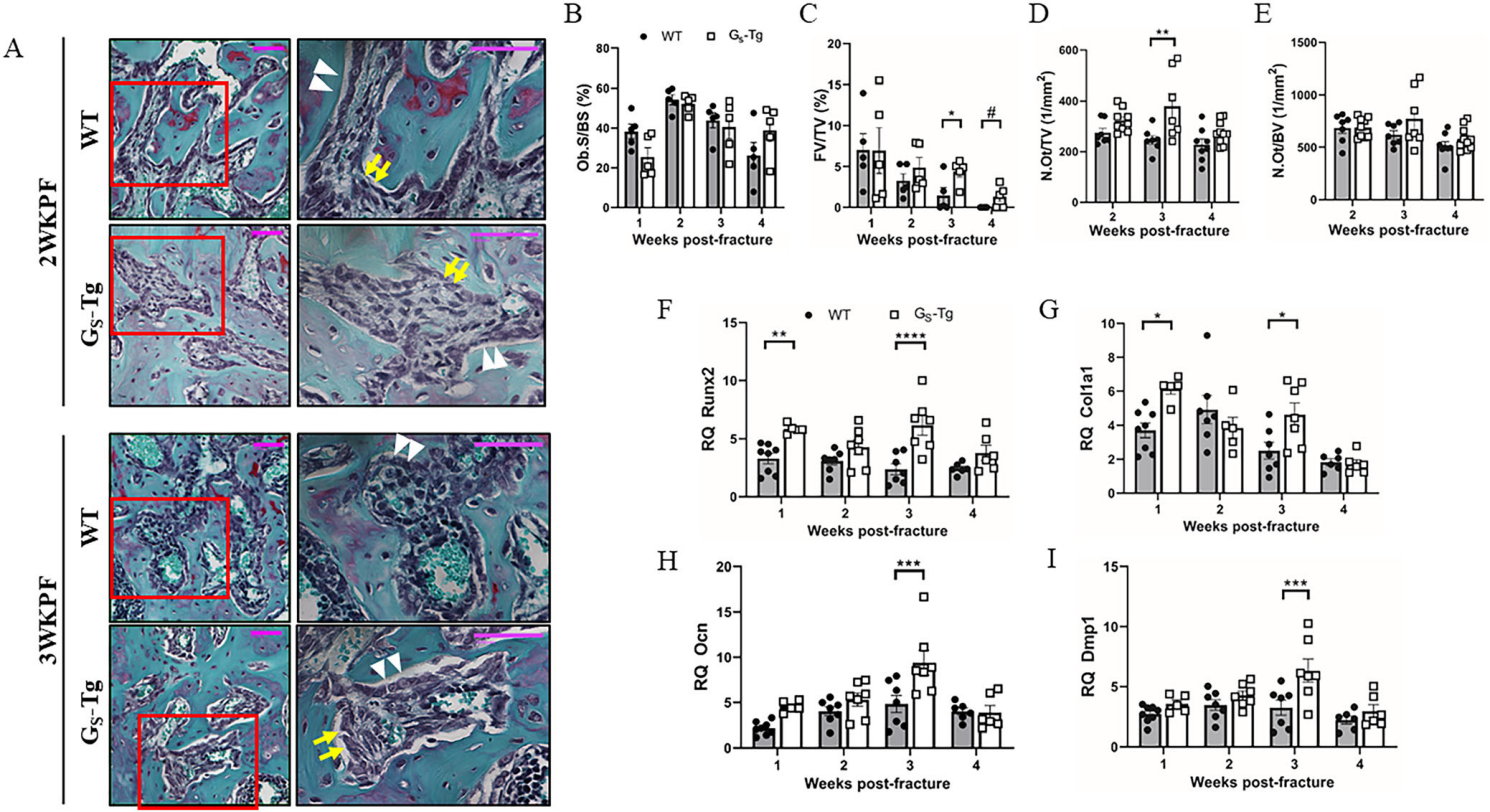
High levels of Gα_S_ increase fibrosis and osteocyte number during bone repair. (*A*) Representative Safranin‐O/Fast Green staining of fractured tibias at 2 and (*B*) 3 weeks post‐fracture at 20× magnification. Scale bar = 50 μm. High‐magnification (40×) images of the selected area enclosed by the red box showing mature osteoblasts (white arrow heads) and fibrosis (yellow arrows). Scale bar = 50 μm. Abundant fibrotic cells line the newly formed woven bone surfaces in G_S_‐Tg mice. (*B*) Histomorphometric quantification of osteoblast surface per bone surface (Ob.S/BS), (*C*) fibrosis volume per tissue volume (FV/TV), (*D*) osteocyte number per tissue volume (N.Ot/TV), and (*E*) per bone volume (N.Ot/BV). *n* = 3 fields of view per section for 5 samples per group. (*F–I*) mRNA expression of osteogenic genes: runt‐related transcription factor 2 (*Runx2)*, type I collagen alpha 1 chain (*Col1a1*), osteocalcin (*Ocn*), and dentin matrix acidic phosphoprotein 1 (*Dmp1*). RT‐qPCR data were normalized to beta 2 microglobulin (*β2M*) expression, and fold changes were expressed relative to the wild‐type (WT) contralateral intact tibia at 1 week post‐fracture. Values represent mean ± SEM. Student's *t* test was used to compare between groups at each time point in *C–F*, and two‐way ANOVA with Sidak's post hoc test was used to detect significant genotype‐specific differences across bone repair in *F–I*. *p < 0.05, **p < 0.01, ***p < 0.001, ****p < 0.0001 compared with WT mice.

Cellular changes underlying robust osteogenesis in G_S_‐Tg fractures were evaluated by histomorphometry and gene expression analysis. The percentage of bone perimeter occupied by osteoblasts (Ob.S/BS) was unchanged in G_S_‐Tg mice compared with WT during the time course of fracture healing (Fig. 3*B*). Instead, increased abundance of fibrotic cells within the newly formed marrow spaces among the woven bone were detected in G_S_‐Tg fractures (Fig. 3*A*, *C*). At 1 week post‐fracture, wild‐type and G_S_‐Tg fractures displayed comparable levels of fibrosis within the callus. However, whereas the amount of fibrosis decreased over time in wild‐type mice, G_S_‐Tg mice persistently exhibited elevated levels of fibrosis until 3 weeks post‐fracture, showing a 67% increase (*p* < 0.05) compared with wild‐type mice. Though reduced by 4 weeks post‐fracture, G_S_‐Tg mice still showed a trend toward increased fibrosis (*p* = 0.058) in the callus relative to wild‐type mice (Fig. 3*C*). Histomorphometry further demonstrated a 54% increase in the number of osteocytes in the callus (N.Ot/TV) at 3 weeks but no difference compared with wild‐type mice once normalized to bone volume (N.Ot/BV) (Fig. 3*D*, *E*), presumably due to increased callus bone volume.

RT‐qPCR analysis revealed upregulation of mRNA encoding osteoblast differentiation and osteocyte markers in the fracture site of G_S_‐Tg mice in parallel with their enhanced ossification. *Runx2*, the master regulator of osteogenesis, was especially highly expressed relative to wild‐type mice with biphasic peak at 1 and 3 weeks post‐fracture (1.8‐fold and 2.6‐fold, respectively) (Fig. 3*F*). The mRNA levels of bone matrix proteins whose expression is activated by *Runx2* were also upregulated. After its maximal induction at 1 week, *Col1a1* expression declined over time but showed a significant upregulation compared with that in wild‐type fractures at 3 weeks (Fig. 3*G*). Conversely, late osteoblast and osteocyte markers *Ocn* and *Dmp1* reached peak expression at 3 weeks (1.9‐fold and 2‐fold higher in G_S_‐Tg, respectively), consistent with osteoblast maturation and increased osteocyte density (Fig. 3*H*, *I*).

The expression of mRNA encoding Gα_S_ in the tibial diaphysis of contralateral bones is increased 2‐ to 2.4‐fold in G_S_‐Tg compared with WT bone. The temporal expression of the Gα_S_ transgene over the time course of bone repair was also examined by RT‐qPCR. Closely paralleling the expression pattern of *Col1a1*, whose full‐length promoter (3.6‐kb Col1a1) was used to drive overexpression of Gα_S_ in osteoblastic cells, *GNAS* was maximally induced at 1WKPF in G_S_‐Tg fractures followed by a progressive decline over time (Fig. 2*F*). The expression patterns of *Col1a1* and *GNAS* are in accordance with the previously reported activity of the 3.6‐kb Col1a1 promoter fragment, demonstrating its broad activity throughout the osteoblast lineage from early osteoblasts and matrix‐synthesizing differentiated osteoblasts.^(^ ^31^ ^)^ The robust induction of *GNAS* in the early reparative phase therefore reflects increased type 1 collagen synthesis by osteoblasts in response to fracture (Fig. 3*G*). Upon transition to the remodeling stage, osteoblastic proliferation likely slowed down over time, consequently decreasing the Gα_S_ transgene induction at later time points.

### High levels of Gα_S_ enhance canonical Wnt signaling and Wnt production in the bony callus

**Fig. 4:**
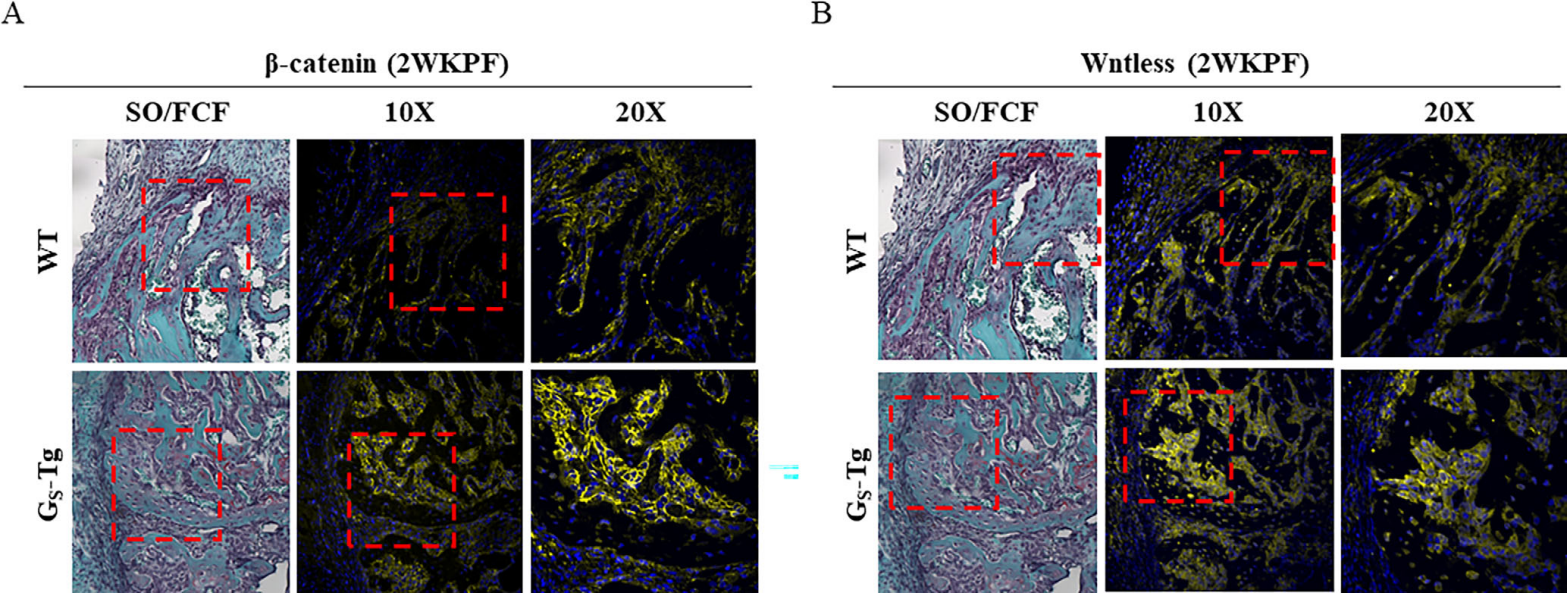
High levels of Gα_S_ enhance Wnt/β‐catenin signaling and Wnts. Representative images of immunofluorescence staining of (*A*) unphosphorylated β‐catenin and (*B*) Wntless (Wls) in serial sections from fracture calluses from wild‐type (WT) and G_S_‐Tg mice at 2 weeks post‐fracture at 10× magnification (middle) with their corresponding Safranin‐O/Fast Green (SO/FCF)‐stained images (left). Boxed regions are shown at higher magnification (20×) at right. Scale bar = 100 μm.

As Wnt/β‐catenin signaling plays a pivotal role in promoting osteoblast differentiation of mesenchymal stem cells and therefore bone formation during development and bone repair, we sought to determine whether Wnt signaling activity is altered by increased Gα_S_ levels during bone repair. Immunofluorescence staining for non‐phosphorylated (active) β‐catenin and Wntless (Wls) was performed on fractures from wild‐type and G_S_‐Tg mice at 2 weeks post‐fracture, where micro‐CT and histology presented the greatest increase in mineralization and bone formation in G_S_‐Tg fractures. Staining of β‐catenin, the key transducer of canonical Wnt signaling, was primarily localized to cells lining the surface of woven bone in both groups but was more intense in fractures from G_S_‐Tg mice, indicating elevated osteoblastic Wnt signaling response (Fig. 4*A*). Likewise, the expression of wntless (Wls), a chaperone protein required for secretion of Wnts, was most readily detected in osteoblastic cells and early osteocytes and was stronger in G_S_‐Tg fractures, suggesting increased osteoblastic Wnt secretion (Fig. 4*B* and Supplemental Fig. S3).

**Fig. 5:**
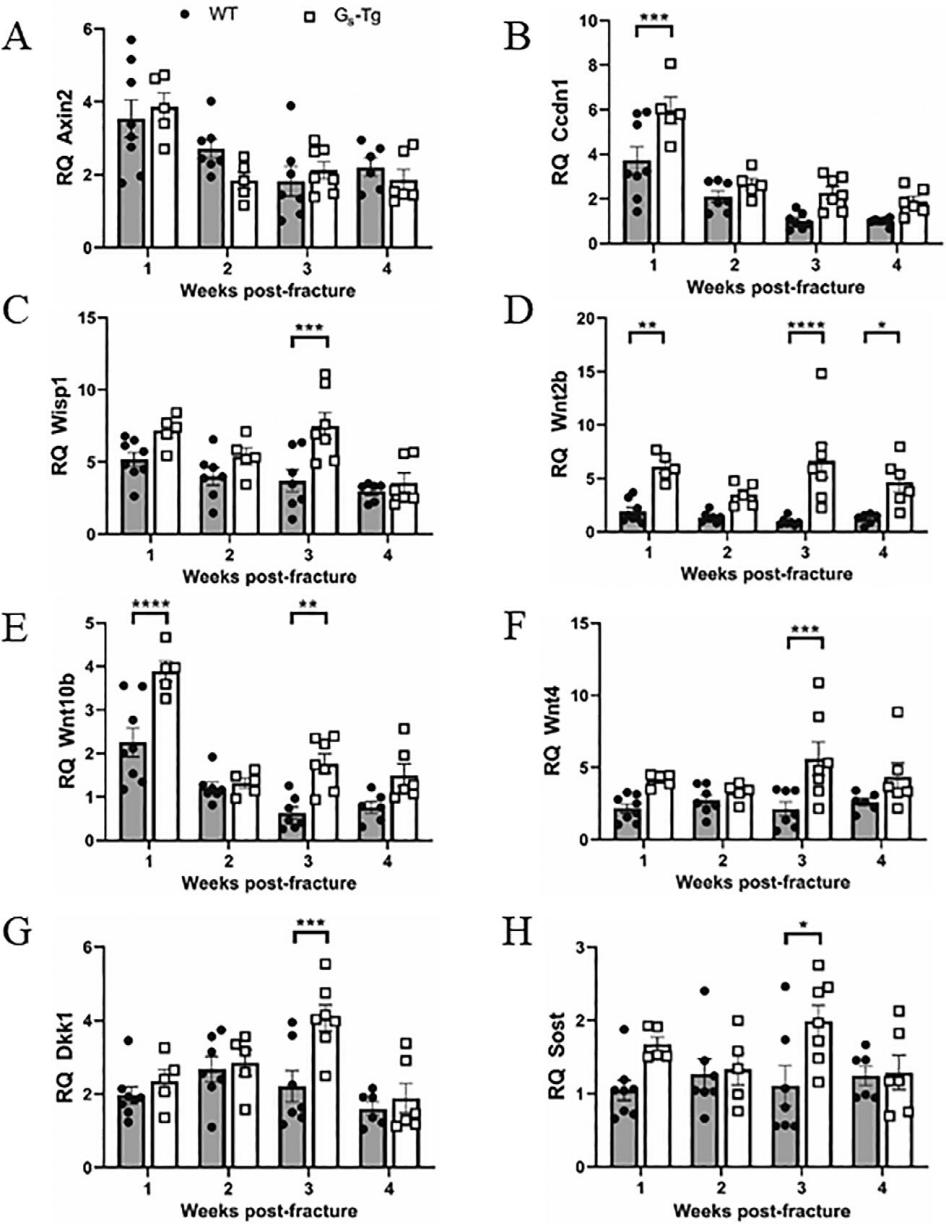
High levels of Gα_S_ upregulate expression of Wnt targets, Wnts, and Wnt antagonists. Temporal gene expression analysis of (*A–C*) Wnt target genes, *Axin2*, *CyclinD1* (*Ccdn1*), and *Wisp1*, (*D–F*) Wnts, *Wnt2b*, *Wnt10b*, and *Wnt4*, and (*G*, *H*) Wnt antagonists, *Dkk1* and *Sost*. RT‐qPCR data were normalized to beta 2 microglobulin (*β2M*) expression, and fold changes were expressed relative to the wild‐type (WT) contralateral intact tibia at 1 week post‐fracture. Values represent mean ± SEM. Two‐way ANOVA with Sidak's post hoc test was used for statistical comparisons. *p < 0.05, **p < 0.01, ***p < 0.001, ****p < 0.0001 compared with WT mice.

The impact of Gα_S_ overexpression on Wnt signaling was further substantiated by temporal mRNA expression of Wnt target genes and Wnts. In comparison to contralateral WT bone, the levels of Wnt target genes *Axin2*, cyclin D1 (*Ccdn1*), and *Wisp1* were all elevated 3‐ to 6‐fold in the fracture calluses of both WT and G_S_‐Tg mice at 1 week post‐fracture (Fig. 5*A–C*). Though no difference in *Axin2* expression was observed between the groups, the downstream targets with known associations to osteoblast proliferation and differentiation, *Ccdn1* and *Wisp1*, were expressed at higher levels with differential timing of peak expressions in G_S_‐Tg fractures (Fig. 5*A–C*). Several canonical Wnts have been shown to be induced during osteogenic events, including bone regeneration after fracture.^(^ ^32^, ^33^ ^)^ The mRNA levels of canonical Wnts, *Wnt2b* and *Wnt10b*, were much more elevated in G_S_‐Tg mice compared with wild‐type mice across bone repair (Fig. 5*D*, *E*). *Wnt2b*, in particular, was elevated up to 7‐fold over levels in wild‐type mice at 3 weeks. In addition, *Wnt4*, which activates both non‐canonical and canonical Wnt signaling pathways, displayed greater expression (up to 3‐fold at 3 weeks) in G_S_‐Tg fractures (Fig. 5*F*). Wnt 5a, which has been shown to stimulate chondrogenesis in early fracture repair,^(^ ^34^ ^)^ was equally upregulated 5‐fold in both WT and G_S_‐Tg fractures at 1 week post‐fracture, the time of highest chondrocyte levels (Supplemental Fig. S4). The expression levels of Wnt antagonists *Dkk1* and *Sost* were upregulated 1.8‐fold in G_S_‐Tg compared with WT at 3 weeks post‐fracture (Fig. 5*G*, *H*). Cumulatively, these data suggest that high levels of G_S_ promote osteogenic response upon bone fracture by increasing osteoblastic Wnts and Wnt signaling.

### Increased osteoclast formation and activity in G_S_‐Tg mice impairs bone remodeling during repair

**Fig. 6:**
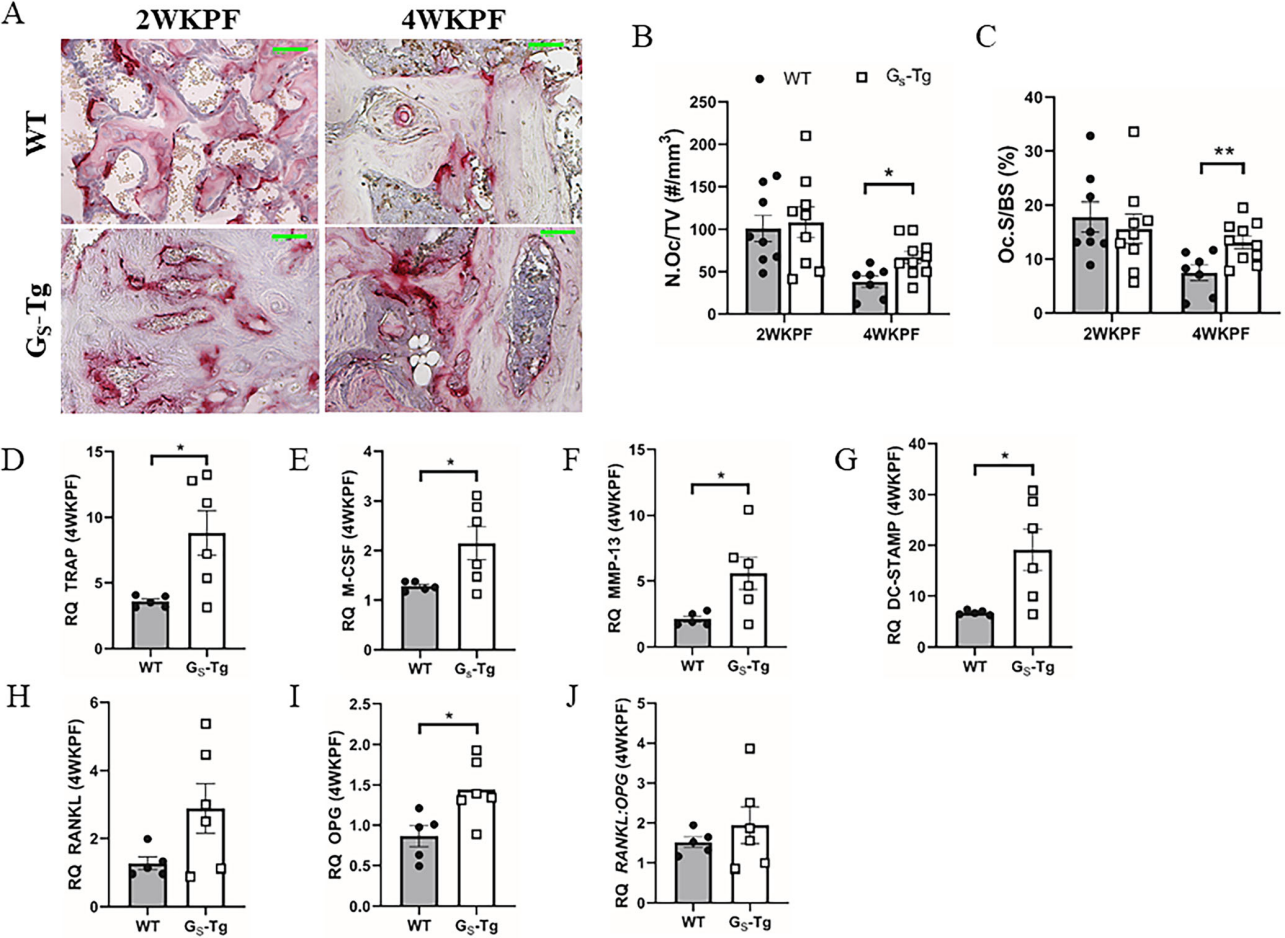
High levels of Gα_S_ elevate osteoclast formation and activity during the bone remodeling phase. (*A*) Representative TRAP‐stained callus sections at 2 and 4 weeks post‐fracture in wild‐type (WT) and G_S_‐Tg mice. Scale bars = 50 μm. (*B*) Quantification of osteoclast number per tissue volume (N.Oc/TV) and osteoclast surface per bone surface (Oc.S/BS). (*C–I*) mRNA expression of osteoclast‐specific genes at 4 weeks post‐fracture: tartrate‐resistant acid phosphatase (*Trap*), macrophage colony‐stimulating factor (*M‐csf*), matrix metalloproteinase 13 (*Mmp‐13*), dendritic cell‐specific transmembrane protein (*Dc‐stamp*), receptor activator of nuclear factor kappa‐B ligand (*Rankl*), osteoprotegrin (*Opg*), and calculated *Rankl:Opg* ratio. RT‐qPCR data were normalized to beta 2 microglobulin (*β2M*) expression and fold changes were expressed relative to the WT contralateral intact tibia. Values represent mean ± SEM. Statistical significance was determined by Student's *t* test and denoted by: *p < 0.05, **p < 0.01 compared with WT mice.

Callus remodeling, which enables conversion of cartilage to bone and remodeling of woven bone to lamellar bone, requires osteoclastic resorption. Osteoclast formation and activity were evaluated by histological examination of TRAP‐stained fracture calluses at 2 and 4 weeks post‐fracture (Fig. 6). At 2 weeks post‐fracture, which coincides with transition to the osteogenic phase of endochondral repair, there were no significant differences in osteoclast parameters between the groups (Fig. 6*A*, *B*). In contrast, by 4 weeks, in which remodeling of woven bone is apparent, abundant and large TRAP+, multinucleated cells were observed in G_S_‐Tg calluses, resulting in 1.8‐fold increases in osteoclast density and size (Fig. 6*A*, *B*). The increase in osteoclastogenesis was confirmed by RT‐qPCR analyses. Expression of a number of osteoclast‐specific genes, including *Trap*, *M‐csf*, *Mmp‐13*, and *Dc‐stamp*, were all robustly upregulated in G_S_‐Tg compared with wild‐type mice (Fig. 6*C–F*). *Rankl* only showed a trend toward increased expression (Fig. 6*G*) and the decoy receptor for Rankl, *Opg*, was also significantly upregulated, which in turn normalized Rankl/Opg ratio (Fig. 6*I*).

### Increased callus mineralization density in G_S_‐Tg mice increases bony callus stiffness

To determine whether the differential fracture healing mechanisms in WT and G_S_‐Tg mice affect the mechanical behavior of the healing bone, torsion tests were performed on fractured tibias at 4 weeks post‐fracture. The geometry‐independent material properties were obtained by normalizing for the dimensions and volume of mineralized bone in the callus derived from micro‐CT scans before torsion testing. The results of micro‐CT scanning and mechanical testing are summarized in Tables 1 and 2. As polar moment of inertia varies with the radial distance from the torsional axis, significant reductions in maximum and minimum radii led to a 1.4‐fold decrease (*p* < 0.05) compared with WT. Despite the smaller callus size, comparable levels of bone formation between WT and G_S_‐Tg mice were reflected in the lack of changes in total mineralized callus volume and cortical thickness and area. Total mineral density (TMD), however, was significantly greater in G_S_‐Tg compared with WT, suggesting formation of more densely mineralized bone in G_S_‐Tg mice. None of the structural properties—yield torque, twist to failure, torsional stiffness, and energy to failure—showed any significant changes between the two groups. However, when normalized to the respective bone geometry, there was a trend toward increased shear stress (*p* = 0.1) and 46% increase in shear modulus (*p* < 0.05), indicating enhanced resistance of the G_S_‐Tg healing bone to stress at the tissue level.

**Table 1:** Micro‐CT Cortical Bone Parameters of Wild‐Type (WT) and Gs‐Tg Fractured Tibias at 4 Weeks Post‐Fracture

	WT (*n* = 9)	G_S_‐Tg (*n* = 8)
TMD (g/cm^3^)	0.63 ± 0.05	0.68 ± 0.05*
TMC (g)	3.51 ± 0.23	3.82 ± 0.70
TMV (mm^3^)	5.63 ± 0.19	5.61 ± 1.19
Cortical thickness (mm)	0.24 ± 0.03	0.24 ± 0.05
Cross‐sectional bone area (mm^2^)	1.52 ± 0.22	1.38 ± 0.21
Maximum radius (mm)	1.30 ± 0.09	1.16 ± 0.10**
Minimum radius (mm)	0.83 ± 0.05	0.73 ± 0.06**
*J*_avg_ (mm^4^)	0.95 ± 0.14	0.67 ± 0.16**

Abbreviations: *J*_avg_ = average polar moment of inertia; TMC = total mineral content (g); TMD = total mineral density (g/cm^3^); TMV = total mineralized tissue volume (mm^3^).

*Note*: Values represent mean ± SD. *p < 0.05 and **p < 0.01 compared with WT mice.

**Table 2:** Torsional Mechanical Properties of Wild‐Type (WT) and G_S_‐Tg Fractured Tibias in 4‐Week Post‐Fracture Mice

	WT (*n* = 9)	G_S_‐Tg (*n* = 8)
Structural properties		
Yield torque (N.mm)	24.41 ± 7.46	24.76 ± 6.91
Angle at failure (°)	11.58 ± 1.65	11.95 ± 2.04
Torsional stiffness (N.mm/°)	2.74 ± 0.76	2.61 ± 0.80
Energy to failure (mJ)	163.23 ± 61.48	174.00 ± 65.03
Material properties		
Shear stress (MPa)	33.77 ± 11.66	43.63 ± 12.33^#^
Shear strain	3.40 ± 0.44	3.06 ± 0.73
Shear modulus (MPa)	10.12 ± 3.71	14.80 ± 4.57*
Toughness (mJ/mm^2^)	24.77 ± 9.43	28.35 ± 11.49

Abbreviation: mJ = millijoules; MPa = megapascals; N.mm = millinewtons.

*Note*: Values represent mean ± SD. *p < 0.05 and ^#^ *p* ≤ 0.1 compared with WT mice.

## Discussion

The normal bone healing cascade fails in 5% to 10% of all fractures, resulting in delayed union or non‐union. Although a number of conditions, such as advanced age, vascular disease, diabetes mellitus, and smoking, impede osteogenesis during bone repair and predispose individuals to malunion,^(^ ^4^, ^35^ ^)^ little is known about the underlying causes of divergent fracture healing responses among healthy individuals. The effect of increased G_S_ expression on the bone phenotype in G_S_‐Tg mice at the time of osteotomy is found primarily in trabecular bone, where we have previously shown a 75% increase in bone volume as a result of increased trabecular number.^(^ ^20^ ^)^ Cortical bone was much less affected with small increases in cortical bone area and thickness; however, there was a large increase in cortical porosity in G_S_‐Tg mice. Thus, it was important to determine response to bone fracture in these mice and the biomechanical strength after fracture repair. Although very much underexplored, healthy individuals do express a large range of G_S_ protein levels in their cells,^(^ ^19^ ^)^ and the present study highlights the significance of variation in osteoblast Gα_S_ levels on endochondral healing.

The endochondral healing response in G_S_‐Tg mice was characterized by suppression of cartilage formation and enhanced bone formation, resembling intramembranous ossification. At 1 week post‐fracture, the time of peak soft callus formation, there was a pronounced decrease in the callus cartilage volume fraction along with downregulation of chondrogenic genes. The expression of *Sox9*, the master regulator of chondrogenesis, however, was less affected than that of more differentiated chondrogenic markers, *Col2a1* and *Col10a1*. Lineage‐tracing studies have shown that *Sox9* expression is not only restricted to chondroprogenitors but also present in osteochondral mesenchymal progenitors that participate in endochondral bone formation.^(^ ^36^, ^37^ ^)^ It is thus likely that the increase in uncommitted mesenchymal progenitors contributed to *Sox9* expression in the G_S_‐Tg callus, resulting in a relatively subtle downregulation.

Based on the opposing effects on cartilage and bone formation, these results demonstrate that the level of Gα_s_ expression is crucial in determining the fate of osteochondral progenitors and that elevated Gα_s_ levels confer an increased osteogenic differentiation potential at the expense of chondrogenic differentiation, driving more rapid new bone formation. This is in keeping with the ability of G_S_ signaling to promote bone formation.^(^ ^8^, ^38^ ^)^ It is possible that chondrocytes formed more rapidly before 1 week post‐fracture in G_S_‐Tg callus and were replaced by osteoblasts by week 1; however, this seems unlikely as osteoblast numbers peaked at the same time in WT and G_S_‐Tg callus at 2 weeks post‐fracture (Fig. 3*B*) along with the persistence of increased fibrous cells in Gs‐Tg. In transgenic mice with osteoblast‐specific Gα_i_ deficiency, there was no effect on chondrogenesis and only a modest effect on osteoblastogenesis during endochondral repair.^(^ ^39^ ^)^ The different effects on fracture repair between this model and G_S_‐Tg mice likely result from Gα_i_ ablation in cells further along the osteoblast pathway with restricted differentiation potential. Since the degree of interfragmentary strain in the fracture callus can also influence the type of fracture repair that occurs with low strain favoring intramembranous healing,^(^ ^27^, ^40^, ^41^ ^)^ we also determined whether there is a difference in the size of the medullary canal and therefore mechanical stability between WT and G_S_‐Tg mice by measuring the volume of the medullary cavity in the contralateral intact bone on micro‐CT images. G_S_‐Tg mice displayed an average medullary cavity that was not significantly different from that of WT mice (Supplemental Fig. S1), confirming that the diminished chondrogenic response is not a consequence of low interfragmentary strain in the healing callus of G_S_‐Tg mice.

At the cellular level, however, we did not observe increases in osteoblasts accompanying increased bone formation at 2 and 3 weeks post‐fracture in G_S_‐Tg mice. Instead, excess fibroblast‐like cells were observed, often in place of osteoblasts, along the newly formed woven bone surfaces, similar to the previously observed increase in fibrosis in the G_S_‐Tg trabecular bone after cPTH treatment.^(^ ^21^ ^)^ These fibroblast‐like cells have been identified as pre‐osteoblasts capable of differentiating into mature osteoblasts; they express osteoblast differentiation markers and upon discontinuation of PTH, mature into matrix‐synthesizing osteoblasts and then osteocytes.^(^ ^42^ ^)^ In agreement with these observations, higher mRNA levels of osteoblast markers were detected concomitant with increased fibrosis in G_S_‐Tg mice during bone healing. These findings suggest that increased Gα_s_ levels induce fibrosis, which in turn serves as a reservoir of osteoblastic cells that can later differentiate into mature osteoblasts and secrete bone matrix. Once differentiated, rapid bone formation prematurely encapsulates osteoblasts, thereby resulting in no observable change in osteoblast abundance. This is evidenced by simultaneous increases in callus bone volume, markers for mature osteoblasts, *Ocn*, and early osteocyte, *Dmp1*, and increased osteocyte density at 3 weeks post‐fracture.

One caveat to our study protocol that might affect fracture healing was the use of meloxicam to control inflammation in the first 3 days after surgery. Studies using cyclo‐oxygenase‐2 (Cox‐2) selective inhibitors and knockout mice have demonstrated the importance of Cox‐2‐dependent prostaglandin synthesis during normal bone repair. These studies highlight that local induction of Cox‐2/prostaglandins is essential in promoting osteogenic differentiation of mesenchymal progenitors and stimulating endochondral and intramembranous bone formation during fracture repair.^(^ ^25^, ^26^ ^)^ However, evidence for negative effects of Cox‐2 inhibition on fracture repair have been mainly from preclinical studies in which Cox‐2 activity was blocked throughout the course of healing. With short‐term use (up to 7 days post‐surgery in rats with mid‐diaphyseal fractures), the inhibitory effects of NSAIDs on the bone repair process were far less apparent, with only a transient delay in mechanical recovery of fractured bone that is later reversed.^(^ ^27^, ^28^ ^)^ Thus it seems likely that 3 days’ exposure to meloxicam would have had even less effect on the outcomes of fracture repair in our mice.

Given the well‐established role of Wnt/β‐catenin signaling as a key driver of bone formation and osteogenic commitment of mesenchymal progenitors during bone repair,^(^ ^32^, ^33^ ^)^ upregulation of β‐catenin signaling may be a mechanism whereby high levels of Gα_S_ increase osteogenesis and stimulate fibrosis during fracture repair. Indeed, IF staining and qPCR data demonstrated increased activation of canonical Wnt signaling and Wnt secretion in G_S_‐Tg mice during bone repair. While canonical Wnt induction regulates osteogenic differentiation, non‐canonical Wnt 5 has been reported to be upregulated during early fracture repair and plays a role in stimulation of chondrogenesis by suppressing canonical Wnt signaling.^(^ ^43^, ^44^ ^)^ Deletion of Wnt 5a in mice resulted in decreased chondrogenesis and delayed fracture healing.^(^ ^34^ ^)^ We found robust upregulation of Wnt5a at 1 week post‐fracture in both strains of mice; however, the more robust stimulation of canonical Wnts 2b and 10b in G_S_‐Tg mice appeared to tip the balance toward osteogenesis. Indeed, expression of Wnt target genes, *Cyclind1* and *Wisp1*, were significantly upregulated in G_S_‐Tg mice, indicating elevated Wnt pathway activity. IF staining of active β‐catenin in the callus during the peak osteo‐anabolic phase of bone repair (2 weeks) further demonstrated that Wnt signaling is predominantly activated in osteoblastic cells but to a greater extent in G_S_‐Tg mice than in wild‐type mice. Consistent with these observations are studies that demonstrate upregulation of the canonical Wnt pathway in bone lesions harboring fibrotic cells in FD mouse models with constitutive Gα_S_ activity in osteolineage cells.^(^ ^6^, ^45^ ^)^ Several canonical Wnts (*Wnt2b*, *4*, and *10b*), all of which have been shown to enhance osteogenic differentiation, were also robustly induced in G_S_‐Tg mice throughout the repair process. In addition, comparatively strong staining of Wntless, a chaperone protein involved in secretion of Wnts, was detected in osteoblastic cells within the bony callus at 2 weeks (Fig. 4*A*, *B*), suggesting that high levels of Gα_S_ enhance Wnt signaling and hence osteogenesis in part by elevating osteoblast‐specific Wnt production. This is in line with other studies showing that PTH treatment, a well‐characterized stimulator of Gα_S_ in osteoblastic cells, upregulates Wnts during fracture repair and increases bone formation.^(^ ^46^ ^)^ Similarly, Gα_S_‐coupled prostaglandin EP2 and EP4 receptors are involved in local Cox‐2 regulation of Wnt/β‐catenin signaling in response to bone loading^(^ ^47^ ^)^ and could mediate the effects of Cox‐2 increasing mesenchymal cell differentiation into osteoblasts in bone repair.^(^ ^25^ ^)^ Collectively, our results suggest that Gα_S_ overexpression initially promotes osteogenic differentiation potential of mesenchymal stem cells (MSCs) via upregulation of canonical Wnt signaling, but its constant stimulation subsequently promotes a fibrotic state of these cells.

We also found discrete stimulation of Wnt inhibitors *Dkk1* and *Sost* specifically at 3 weeks post‐fracture when osteocytes are elevated in G_S_‐Tg mice. Similar increases in Wnt antagonists were reported in mice treated with PTH 1‐34 during the hard‐callus phase of bone repair.^(^ ^11^, ^48^ ^)^ Since the expression of both of these genes is suppressed by the cAMP/PKA pathway,^(^ ^48^, ^49^ ^)^ their induction in G_S_‐Tg bone seems paradoxical. The upregulation may be part of a negative feedback mechanism in response to heightened activation of Wnt signaling in G_S_‐Tg osteoblasts. Several studies have provided evidence that timely downregulation of canonical Wnt signaling during osteoblast differentiation is essential for proper osteoblast maturation and bone matrix production and mineralization, and constitutive activation impairs bone regeneration during fracture repair. In support of this, van der Horst and colleagues showed in vitro that Wnt antagonists are sharply upregulated in differentiated osteoblasts.^(^ ^50^ ^)^ There is also the possibility that other regulatory factors are involved in controlling the expression of Wnt inhibitors in bone repair. For example, Osx, an osteogenic transcription factor that acts downstream of Runx2, can activate the promoters of *Sost* and *Dkk1*.^(^ ^51^, ^52^ ^)^ Furthermore, *Dkk1* itself is a target gene of β‐catenin‐mediated signaling.^(^ ^53^ ^)^ Although *Osx* was not examined in our study, *Runx2*, *Wnt2b*, and *Wnt4* expression were all maximal at 3 weeks post‐ fracture and highly increased in G_S_‐Tg, suggesting potential alternate pathways for stimulation of *Sost* and *Dkk1* in these mice.

By 4 weeks post‐fracture, attenuation of the robust anabolic response in G_S_‐Tg mice was evidenced by loss of differences in bone formation indices, such as mineralized tissue volume, histological bone volume, and cross‐sectional area, in comparison to WT. Although the progressive decline in *GNAS* transgene expression with advancement of fracture healing provides a plausible explanation for comparatively decreased osteogenesis and thus normalization of mineralized tissue quantity in 4‐week‐old calluses, elevated osteoclast formation and activity at 4 weeks point to enhanced osteoclastic callus remodeling as another contributing factor. Increased TRAP staining and induction of osteoclast differentiation factors only at the later stage of repair suggest that osteoclasts in G_S_‐Tg fractures are elevated in response to stimulation by osteoblastic cells and profoundly greater mineralized callus at an earlier time point. The presence of osteoclast‐lined pores in G_S_‐Tg cortical bone is the primary effect of increased G_S_ on this bone compartment.^(^ ^20^ ^)^ The presence of increased osteoclasts at 4 weeks post‐fracture in G_S_‐Tg bone indicates recapitulation of this basal phenotype in the later stages of fracture repair.

Despite the apparent difference in ossification between WT and G_S_‐Tg mice and elevated osteoclasts in G_S_‐Tg mice, the results of biomechanical tests showed comparable structural mechanical properties in both groups, presumably because of restoration of the callus bone morphology to WT levels at 4 weeks post‐fracture. A significant increase in shear modulus, along with a non‐significant increase in shear stress and decrease in shear strain, however, was indicative of enhanced material stiffness and thus higher‐quality bone in G_S_‐Tg mice. The observed increase can be explained by reduced polar moment of inertia in G_S_‐Tg mice, since shear modulus is inversely related to polar moment of inertia. Bone stiffness has also been shown to be greatly influenced by the degree of bone mineralization; several studies have described a direct relationship between shear modulus, a material index of stiffness, and bone mineral density.^(^ ^54^, ^55^ ^)^ In accordance with this, the increase in shear modulus was accompanied by increased total mineral density in the G_S_‐Tg callus, suggesting that tissue‐level variation in mineral content underlies the difference in callus stiffness between WT and G_S_‐Tg mice.

In summary, the present study demonstrated that high levels of Gα_S_ redirect the fracture‐healing response to favor osteogenesis over chondrogenesis, thereby resulting in increased fibrosis and new bone formation at the expense of cartilage formation. Such alterations in the repair process improve the biomechanical competence of healing bone owing to increased material stiffness. We also report that one of the molecular responses to increased Gα_S_ levels is increased osteoblast‐specific production of Wnts and activation of canonical Wnt signaling. This is the first demonstration that the mode of bone healing can be modulated solely by alterations in normal Gα_S_ levels. These findings may offer insight into the management and treatment of bone fractures as the osteoanabolic drugs that show therapeutic potential in fracture healing, namely PTH and romosozumab, mediate their stimulatory effects either via Gα_S_ signaling to increase Wnts or modulation of sclerostin, both leading to increased canonical Wnt signaling.

## Author Contributions

**Jane Mitchell:** Conceptualization; formal analysis; funding acquisition; project administration; writing – review and editing. **Kathy K Lee:** Data curation; formal analysis; investigation; methodology; writing – original draft. **Adele Changoor:** Methodology; supervision; writing – review and editing. **Marc D Grynpas:** Funding acquisition; methodology; supervision; writing – review and editing.

### Peer Review

The peer review history for this article is available at https://www.webofscience.com/api/gateway/wos/peer-review/10.1002/jbm4.10841.

## Disclosures

The authors have no conflicts to disclose.

## Supporting information


**Fig. S1.** X‐ray images of WT and Gs‐Tg tibias at 1 week post‐fracture.

**Fig. S2.** Medullary cavity volume measured in contralateral, unfractured tibias of WT and Gs‐Tg mice at 1 weeks post‐fracture.

**Fig. S3.** Additional images of unphosphorylated β‐catenin and Wls immunofluorescence staining in fracture calluses from WT and Gs‐Tg mice at 2 weeks post‐fracture. Scale bars = 100 μm.

**Fig. S4.** RT‐qPCR analysis of mRNA extracted from fracture calluses of WT and G_S_‐Tg mice at 1WKPF shows no difference Wnt5a expression.

**Table S1.** Primer sequences used in real‐time PCR.

## Data Availability

The data that support the findings of this study are available from the corresponding author upon reasonable request.
