# Supplementary figures and images for: Increased Osteoblast GαS Promotes Ossification by Suppressing Cartilage and Enhancing Callus Mineralization During Fracture Repair in Mice
Source: JBMR Plus. 2023 Nov 15;7(12):e10841. doi: 10.1002/jbm4.10841 (PMC10731140; doi:10.1002/jbm4.10841)

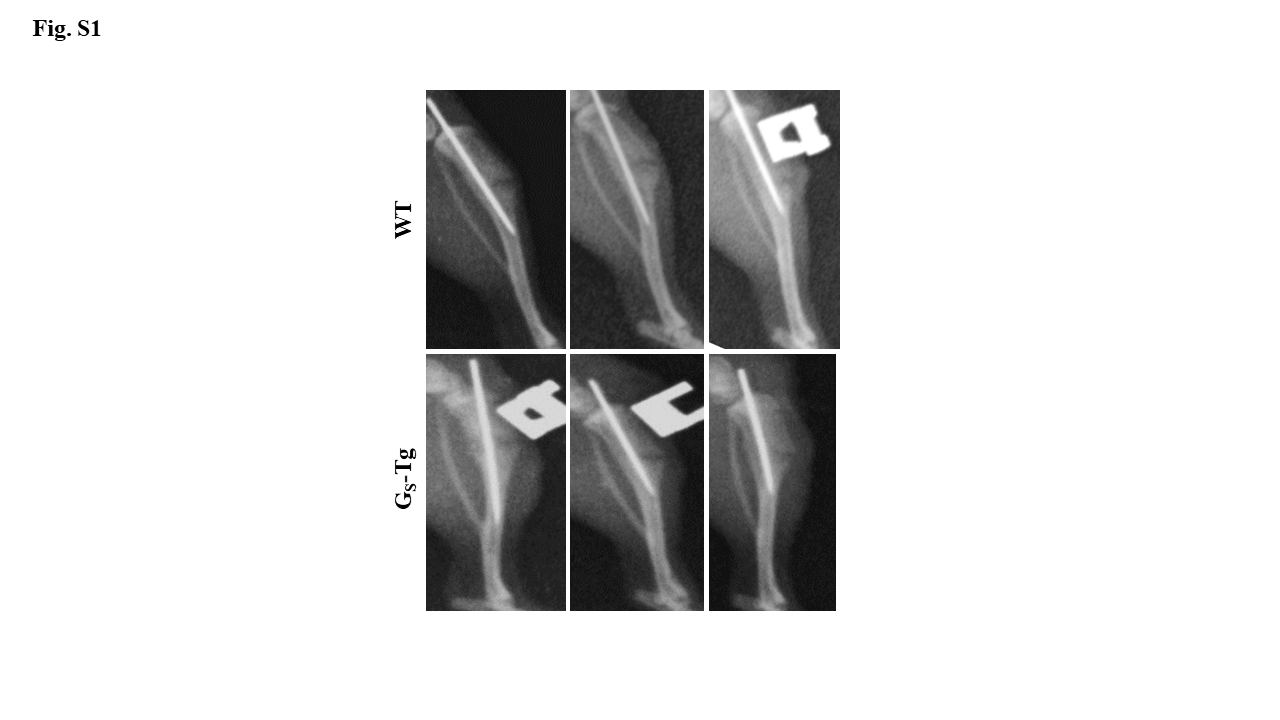

Supplement: Supplementary file 1 — Fig. S1. X‐ray images of WT and Gs‐Tg tibias at 1 week post‐fracture. [file JBM4-7-e10841-s003.tiff]

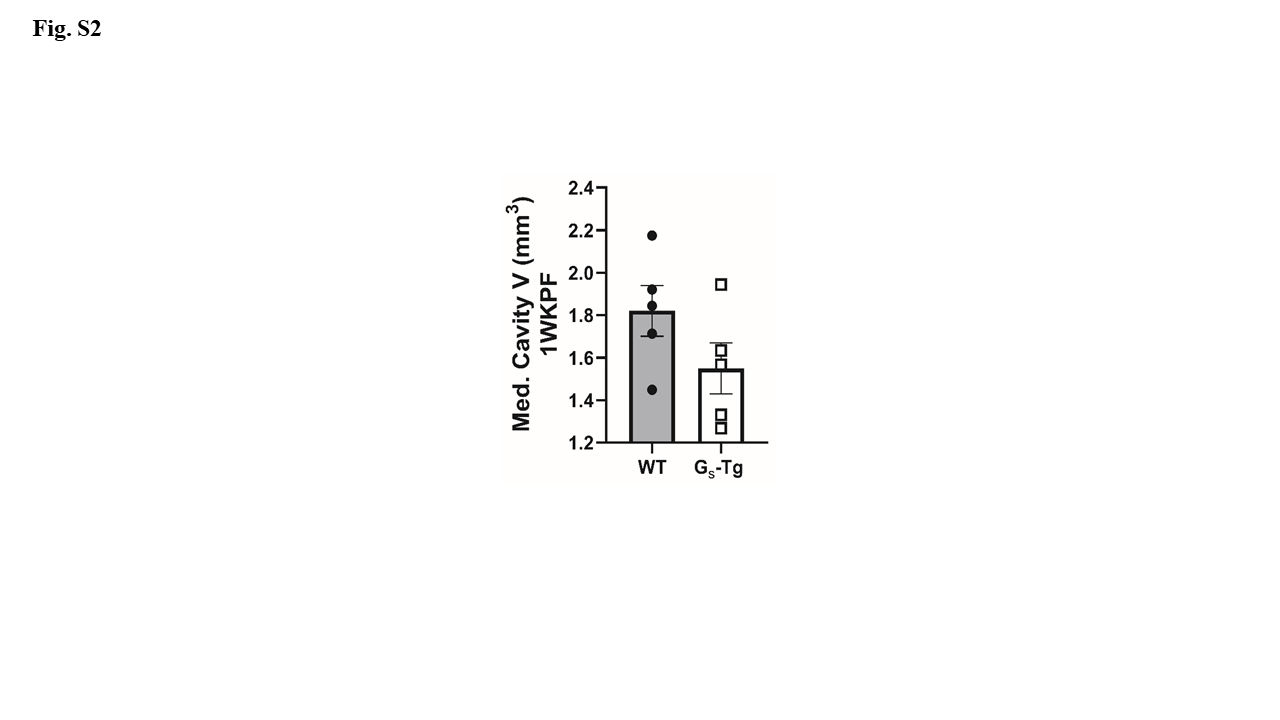

Supplement: Supplementary file 2 — Fig. S2. Medullary cavity volume measured in contralateral, unfractured tibias of WT and Gs‐Tg mice at 1 weeks post‐fracture. [file JBM4-7-e10841-s001.tiff]

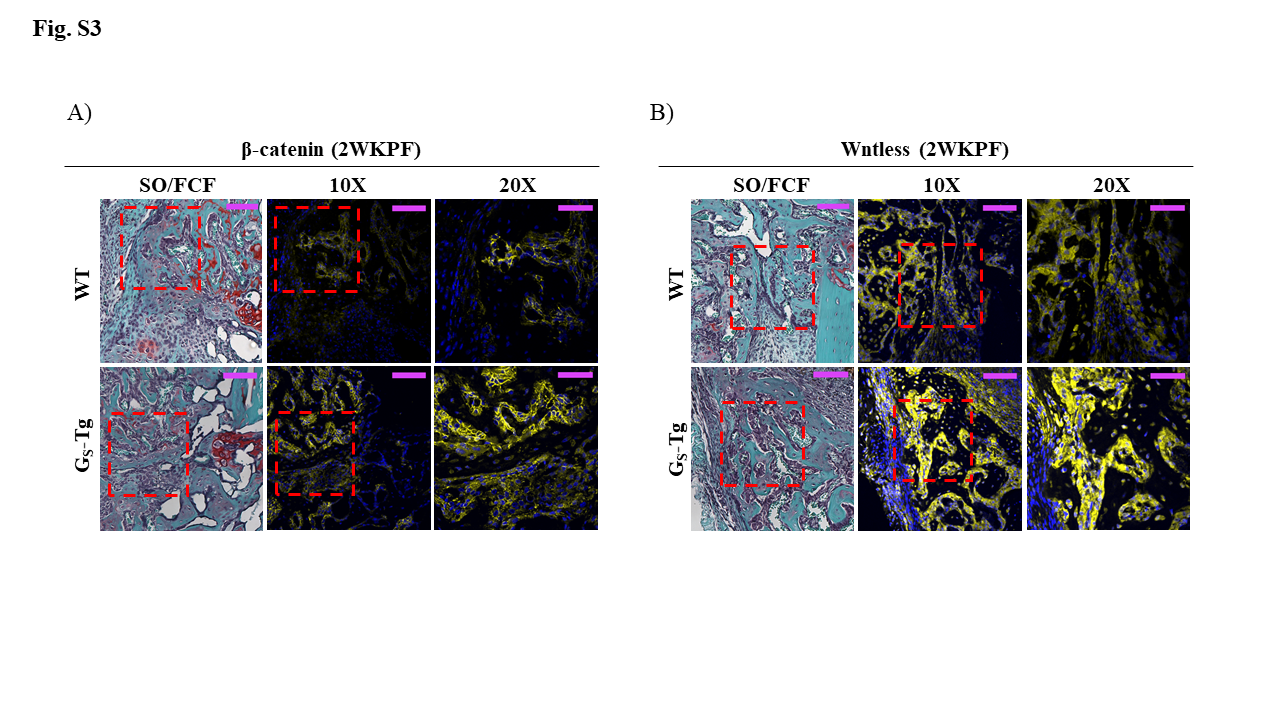

Supplement: Supplementary file 3 — Fig. S3. Additional images of unphosphorylated β‐catenin and Wls immunofluorescence staining in fracture calluses from WT and Gs‐Tg mice at 2 weeks post‐fracture. Scale bars = 100 μm. [file JBM4-7-e10841-s005.tiff]

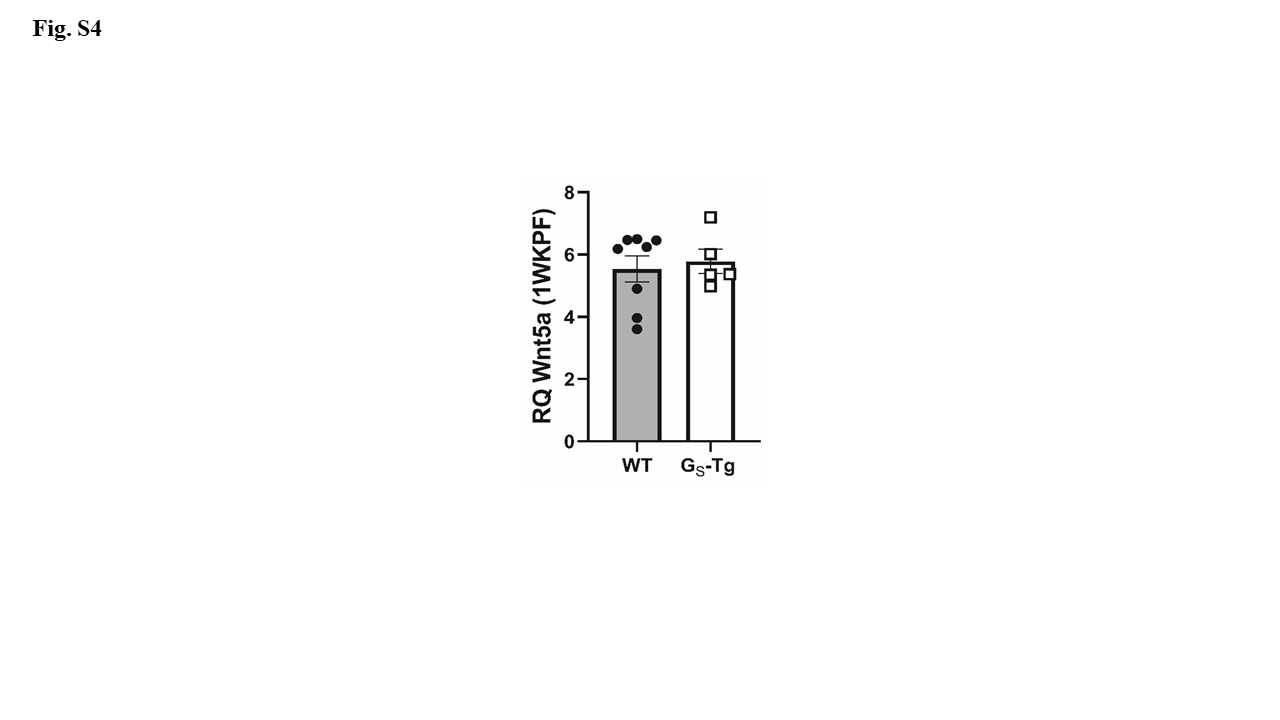

Supplement: Supplementary file 4 — Fig. S4. RT‐qPCR analysis of mRNA extracted from fracture calluses of WT and GS‐Tg mice at 1WKPF shows no difference Wnt5a expression. [file JBM4-7-e10841-s002.tiff]
