# Supplementary material for: Increased Osteoblast GαS Promotes Ossification by Suppressing Cartilage and Enhancing Callus Mineralization During Fracture Repair in Mice
Source: JBMR Plus. 2023 Nov 15;7(12):e10841. doi: 10.1002/jbm4.10841 (PMC10731140; doi:10.1002/jbm4.10841)
Supplement: Supplementary file 5 — Table S1. Primer sequences used in real‐time PCR. [file JBM4-7-e10841-s004.docx]

**Supplemental Table S1.** Primer sequences used in real-time PCR

| mRNA | Forward Primer | Reverse Primer |
| --- | --- | --- |
| ***GNAS/Gnas*** | 5’GGCCAACAAAAAGATCGA GAAG3’ | 5’TGCGTGGCCCGGTAGA3’ |
| ***β2M*** | 5’ACCGTCTACTGGGATCGAGA3’ | 5’TGCTATTTCTTTCTGCGTGCAT3’ |
| ***Sox 9*** | 5’ACCCACCACTCCCAAAACC3’ | 5’CGCCCCTCTCGCTTCAG3’ |
| ***Col2a1*** | 5’CCAGGGCTCCAATGATGTAGA3’ | 5’TGTTTCGTGCAGCCATCCT3’ |
| ***Col10a1*** | 5’CCCTGGTTCATGGGATGTTTTATG3’ | 5’TGCCTTGTTCTCTCTTACTG3’ |
| ***Runx2*** | 5’ GCGGTGCAAACTTTCTCCAG3’ | 5’ AGCACTCACTGACTCGGTTG3’ |
| ***Col1a1*** | 5’GCCTTGGAGGAAACTTTGCTT3’ | 5’GCACGGAAACTCCAGCTGAT3’ |
| ***Ocn*** | 5’CTGACAAAGCCTTCATGTCCAA3’ | 5’GGTAGCGCCGGAGTCTGTT3’ |
| ***Dmp1*** | 5’TGTCATTCTCCTTGTGTTCCTTTG3’ | 5’AGAGCTTTCAGATTCAGTATTGTG GTAT3’ |
| ***Rankl*** | 5’GGCCACAGCGCTTCTCA3’ | 5’CCTCGCTGGGCCACATC3’ |
| ***Opg*** | 5’CCACAATGAACAAGTGGC TGTGCT3’ | 5’AGGGTTTCCTGGGTTGTC CATTCA3’ |
| ***M-csf*** | 5’TTGCCAAGGAGGTGTCAGAAC3’ | 5’AAAGGCAATCTGGCATGAAGTC3’ |
| ***Mmp-13*** | 5’CAGTTCCAAAGGCTACAA CTTGTTT3’ | 5’GTCCTTGGAGTGATCCAG ACCTA3’ |
| ***Dc-stamp*** | 5’GGAACCTAAGCGGAACTTAGACA3’ | 5’TAGGGCTTCGTGGAAACACA3’ |
| ***Trap*** | 5’CCACTACCCCATCTGGTCCAT3’ | 5’AAGATACTGCAGGTTGTGGTCATG3’ |
| ***Axin2*** | 5’CCATCTACGCTACTGTCCGTCAT3’ | 5’CGCCAACGACAGCGAGTTAT3’ |
| ***Ccdn1*** | 5’GCGTACCCTGACACCAATCTC3’ | 5’CTCCTCTTCGCACTTCTGCTC3’ |
| ***Wips1*** | 5’TGACTTCCAGGCATGAGGTG3’ | 5’CTAGTGGTGCTGGGGTGAAG3’ |
| ***Wnt2b*** | 5’GACACGTCCTGGTGGTACATAGG3’ | 5’TGGGTAGCGTTGACACAACTG3’ |
| ***Wnt10b*** | 5’TGGGACGCCAGGTGGTAA3’ | 5’CTGACGTTCCATGGCATTTG3’ |
| ***Wnt4*** | 5’CCGGCGCTGGAACTGTT3’ | 5’GTCACCACCTTCCCAAAGACA3’ |
| ***Wnt 5a*** | 5'GGTGCCATGTCTTCCAAGTTC -3' | 5'CCTAGAGACCACCAAGAATTAGCT TCT3' |
| ***Wls*** | 5’ AAAGAAAGGGGGTCGCAGAA3’ | 5’ TGGGTGCTGGAGCGATCA3’ |
| ***Dkk1*** | 5’CCGGGAACTACTGCAAAAAT3’ | 5’CGTTGTGGTCATTACCAAGG3’ |
| ***Sost*** | 5’CGCCAAAGATGTGTCCGAGTA 3’ | 5’TGTCAGGAAGCGGGTGTAGTG3’ |
